# Learning Numerosity Representations with Transformers: Number Generation Tasks and Out-of-Distribution Generalization

**DOI:** 10.3390/e23070857

**Published:** 2021-07-03

**Authors:** Tommaso Boccato, Alberto Testolin, Marco Zorzi

**Affiliations:** 1 Department of General Psychology, University of Padova, Via Venezia 8, 35131 Padova, Italy; tommaso.boccato@studenti.unipd.it; 2 Department of Information Engineering, University of Padova, Via Gradenigo 6, 35131 Padova, Italy; 3 IRCCS San Camillo Hospital, Via Alberoni 70, 30126 Venice-Lido, Italy

**Keywords:** deep neural networks, attention mechanisms, density estimation, numerosity perception, cognitive modeling

## Abstract

One of the most rapidly advancing areas of deep learning research aims at creating models that learn to disentangle the latent factors of variation from a data distribution. However, modeling joint probability mass functions is usually prohibitive, which motivates the use of conditional models assuming that some information is given as input. In the domain of numerical cognition, deep learning architectures have successfully demonstrated that approximate numerosity representations can emerge in multi-layer networks that build latent representations of a set of images with a varying number of items. However, existing models have focused on tasks requiring to conditionally estimate numerosity information from a *given image*. Here, we focus on a set of much more challenging tasks, which require to conditionally generate synthetic images containing a *given number* of items. We show that attention-based architectures operating at the pixel level can learn to produce well-formed images approximately containing a specific number of items, even when the target numerosity was not present in the training distribution.

## 1. Introduction

In recent years, there has been a growing interest in the challenging problem of unsupervised representation learning [1]. Compared to the first wave of supervised deep learning success [2], unsupervised learning has great potential to further improve the capability of artificial intelligence systems, since it would allow building high-level, flexible representations without the need of explicit human supervision. Unsupervised deep learning models are also plausible from a cognitive [3] and biological [4] perspective, because they suggest how the brain could extract multiple levels of representations from the sensory signal by learning a hierarchical generative model of the environment [5,6,7,8].

Early approaches based on deep belief networks [9] already established that unsupervised representation learning leads to the discovery of high-level visual features, such as object parts [10] or written shapes [11,12]. However, the full potential of deep generative models was revealed by the introduction of variational autoencoders (VAE) [13] and generative adversarial networks (GAN) [14], which can discover and factorize extremely abstract attributes from the data [15,16]. These architectures can be further extended to promote the emergence of even more disentangled representations, such as in beta-VAE [17] and InfoGAN [18], or can exploit attention mechanisms to produce meaningful decompositions of complex visual scenes [19].

An interesting case study to investigate the representational capability of deep learning models is that of *numerosity perception*, which consists of rapidly estimating the number of objects in a visual scene without resorting to sequential counting procedures [20]. Compared to other high-level visual features, numerosity information is particularly challenging to extract because it refers to a global property of the visual scene, which co-varies with many other non-numerical visual features such as cumulative area, density and item size [21]. The emergence of numerosity representations has been successfully simulated using deep belief networks [22,23,24], which can approximately estimate the number of items in a given image (matching human-level performance) and partially disentangle it from non-numerical magnitudes [25]. However, learning fully disentangled representations of numerosity seems to be still out of reach even for state-of-the-art generative models, such as the InfoGAN [26].

In this paper, we investigate whether the deployment of *self-attention mechanisms* allows more precisely encoding numerosity information as a disentangled factor of variation. Attention mechanisms [27] were first introduced in the context of machine translation to overcome the limitations of sequence-to-sequence architectures [28], which aimed at compressing the information contained in temporal sequences into fixed-length latent vectors. Shortly after, a novel architecture based solely on attention called *Transformer* [29] achieved new heights by completely dropping recurrence and convolutions. In analogy with the dynamics of associative memories [30], the power of this approach lies in the possibility of using a global attention mechanism to precisely and adaptively weight the contribution of each input element during processing. Transformers are starting to also be applied outside the language domain, with notable success in challenging computer vision tasks [31,32,33].

These promising results motivated the present work, whose main goal is to demonstrate that attention mechanisms can be successfully exploited to learn disentangled representations of numerosity, which can be used to generate novel synthetic images approximately containing a given number of items. Inspired by recent approaches that evaluated the capability of deep generative models to create novel attributes and their combinations [34], the Transformer was probed in different generative scenarios requiring to produce specific numerosities that were never encountered during training. The internal structure of the representational code was also analyzed, in order to investigate whether numerosity information could be mapped into a lower dimensional space that preserves the semantics of cardinal numbers [35].

## 2. Methods

### 2.1. Problem Formulation

Let $D=\{\left(x_{1},n_{1}\right),\ldots ,\left(x_{m},n_{m}\right)\;s.t.\;\left(x,n\right)∼p\left(x,n\right),\;n\in N\}$ be a training dataset consisting of images paired with their respective numerosity (i.e., the number of items contained in each image). The generic (x,n) tuple is sampled i.i.d. from the p(x,n) joint probability mass function (PMF), with N⊂N. The goal is to model the p(x|n) conditional PMF, exploiting a density estimation algorithm which relies solely on global attention applied to the raw input images. Modeling such density by disentangling numerosity from other factors of variation should ideally allow generating images with a controlled number of objects, which could be specified by manipulating the initial state of the generative process (although the generative model does not receive explicit knowledge about cardinal numbers during training, the initial state of the generative process is the same for all training images featuring the same numerosity, as explained in Section 2.2). Crucially, the generative model might even learn to produce out-of-distribution samples belonging to areas of the p(x,n) support that are not represented in D, that is, images containing a number of objects that was never experienced during training.

Practically, one could focus on an equivalent representation of the target density, which exploits the chain rule to allow the density estimation algorithm to work autoregressively:

(1) $$p\left(x|n\right)=\prod_{i=1}^{r}p\left(x_{i}|x_{1},\ldots ,x_{i-1},n\right);$$

where $x=(x_{1},\ldots ,x_{r})$ represents a flattened image x made by *r* pixels. Let $q(x|n,\theta^{⋆})$ be the approximated conditional PMF produced by the density estimation algorithm, with $\theta^{⋆}$ denoting the optimal model parameters; it originates from the minimization of the following negative log-likelihood:

(2) $$\begin{array}{cc} L(\theta ) & =E_{(x,n)∼D}\left[-\log q(x|n,\theta )\right] \end{array}$$

(3) $$\begin{array}{cc} \; & \;\;\;\;\;=E_{(x,n)∼D}\left[-\log\prod_{i=1}^{r}q\left(x_{i}|x_{1},\ldots ,x_{i-1},n,\theta \right)\right] \end{array}$$

(4) $$\begin{array}{cc} \; & \;\;\;\;\;=E_{(x,n)∼D}\left[-\sum_{i=1}^{r}\log q\left(x_{i}|x_{1},\ldots ,x_{i-1},n,\theta \right)\right]. \end{array}$$

Step (4) suggests that the Transformer can be straightforwardly trained by computing the CrossEntropyLoss criterion on the model output logits exploiting PyTorch [36].

### 2.2. Model Architecture

The model investigated in this work is an encoder-only Transformer capable of dealing with data characterized by spatial relationships (e.g., images); its backbone, indeed, is built from the self-attention layers devised in [29]. Overall, the following mapping is implemented: $\left(x,n\right)\mapsto P\in R^{q\times p}$, where $x=(x_{1},\ldots ,x_{q})$ denotes the categorical input intensities, q≤r and $p_{i}^{T}$ (i.e., the *i*th row of P) represents the conditional PMF associated to $x_{i}$ (the density support, of cardinality *p*, coincides with the set of input intensities).

Input frames are not directly fed into the Transformer encoder: pixel intensities are first scanned following the raster order, and then transformed into learnable embeddings to which the position information is added. Being the positional encodings also learned, it is important to highlight that the model is invariant with respect to the order in which inputs are supplied; however, once the order is fixed, it must be maintained. During the last processing stage, the encoder output goes through a linear layer. Hence, the conditional probability mass functions (PMFs) are computed by applying a softmax function to the produced logits.

The deployed encoder only accepts sequences of real-valued *d*-dimensional vectors. As a consequence, the supplied dataset entries undergo careful processing. Firstly, the $\left(x_{1},\ldots ,x_{q-1}\right)$ intensities are mapped into q−1 embeddings (pixel $x_{q}$ is never consumed by autoregression), $x\in R^{(q-1)\times d}$. Then, the encoder input is computed as:

(5) $$H_{0}={\left[s,x^{T}\right]}^{T}+E;$$

where $s\in R^{d}$ encodes the equivalence class to which the considered image belongs (i.e., the numerosity *n*) while $E\in R^{q\times d}$ stores information about the pixel positions. Borrowing the machine translation nomenclature, we call s the *Start of String* (SoS). Input embeddings, SoSs and positional encodings are obtained in the same way: the discrete starting values (i.e., intensities, numerosities and positions) trivially become indexes capable of selecting the corresponding rows in one of the $W_{x}\in R^{p\times d}$, $W_{s}\in R^{|N|\times d}$ and $W_{E}\in R^{r\times d}$ matrices, learned through backpropagation [31,32,33]. We emphasize that the learned embeddings minimize the introduction of explicit inductive biases: the untrained model, indeed, is completely unaware of the distances between gray levels, the ordering on N and the correlations of pixels. As a side effect, $W_{E}$ constrains the input image resolution.

The encoder consists of 2L properly stacked multi-head scaled dot-product attention (mha(•)) and point-wise fully connected (fc(•)) sub-layers. Residual connections and layer normalizations (norm(•)) complete the architecture. Resuming from (5),

(6) $$\begin{array}{cc} A_{l} & =\mathrm{norm}(H_{l-1}+\mathrm{mha}\left(H_{l-1}\right)) \end{array}$$

(7) $$\begin{array}{cc} H_{l} & =\mathrm{norm}(A_{l}+\mathrm{fc}\left(A_{l}\right)) \end{array}$$

describe the encoder pipeline, with the l∈[1,L] subscript denoting the considered layer. The detailed implementations of mha(•), fc(•) and norm(•) can be found in [29]. Finally, the linear(•) and softmax(•) functions are assembled to produce the target conditional densities:

(8) $$P=\mathrm{softmax}(\mathrm{linear}\left(H_{L}\right)).$$

**Figure 1:**
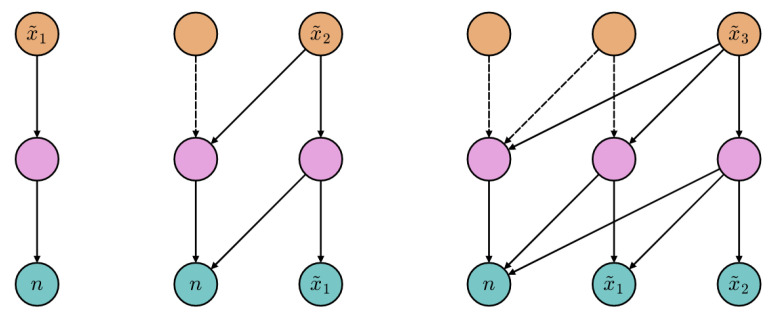
Example attention graphs (L=2, r=3) describing the *spontaneous generation* (see Section 2.4) of a novel image. Teal, pink and orange nodes represent the input, hidden and output positions, respectively. For each query node, the outgoing connections indicate which positions can be adaptively weighted.

The *attention graphs* [37] shown in Figure 1 help us in explaining how the encoder autoregression is achieved. Directed edges identify the allowed attention flows; the missing ones (with respect to the respective fully connected, bipartite sub-graphs) are masked to prevent queries from attending to illegal positions. Solid edges denote the active attention flows involved in the generation of the considered gray level. The generative loop starts as in the left graph, where the represented forward pass results in the sampling of the first intensity, ${\tilde{x}}_{1}$. In the middle and right graphs, the gray level obtained during the previous pass is appended to the input sequence, and the process is repeated. Further details about the model architecture and training hyperparameters are reported in Appendix A.

### 2.3. Datasets

The Transformer was trained on two different datasets containing images of size 32×32 pixels with a varying number of objects (white dots) placed on a black background. Numerosities were uniformly sampled from the set {1,…,8}. Each dataset was split into training, validation and test subsets containing, respectively, 16,000, 3200 and 3200 images. We verified that the size of the training set was properly calibrated by plotting the validation loss as a function of the number of training patterns (see Figure A2).

**Figure 2:**
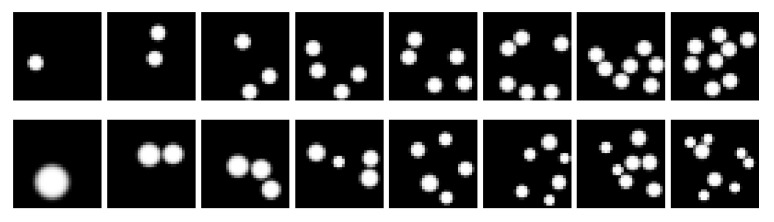
Sample images from the two datasets considered. Stimuli with increasing numerosity (i.e., N={1,…,8}) are progressively shown from left to right. The top row contains samples from the *Uniform Dots* dataset, where the rendered dots have the same radius. The bottom row contains samples from the *Non-Uniform Dots*, where the area of each dot is sampled from $N(\mu_{frame}/n,\sigma_{dot}^{2})$.

The first dataset, which we call *Uniform Dots*, contained images featuring objects of uniform size (see samples in the top row of Figure 2). In this dataset, the numerosity information is perfectly correlated with the total number of active pixels, which does not allow assessing to what extent the Transformer can disentangle numerosity from cumulative area. We thus also introduced a second dataset, which we call *Non-Uniform Dots*, containing images featuring objects of different size and constant (on average) cumulative area (see samples in bottom row of Figure 2). Let $A_{dot}∼N\left(\mu_{dot},\sigma_{dot}^{2}\right)$ be the random variable quantifying the individual area covered by a dot. The total area covered in a frame characterized by *n* dots can be expressed as:

(9) $$\begin{array}{cc} A_{frame} & =\sum_{i=1}^{n}A_{dot} \end{array}$$

(10) $$\begin{array}{cc} \; & =nA_{dot} \end{array}$$

(11) $$\begin{array}{cc} \; & ∼N(n\mu_{dot},n^{2}\sigma_{dot}^{2}). \end{array}$$

Setting $\mu_{dot}=\frac{\mu_{frame}}{n}$ implies that $A_{frame}∼N\left(\mu_{frame},n^{2}\sigma_{dot}^{2}\right)$, thus making the expected cumulative area $E\left[A_{frame}\right]=\mu_{frame}$ independent from *n* (in our case, these parameters were set to $\mu_{frame}=150$ and $\sigma_{dot}=8$).

### 2.4. Generative Tasks

To investigate the emergence of numerosity representations, we designed a variety of generation tasks. The objective of these experiments was twofold: on the one hand, they allowed establishing whether the learned representations could be used to produce synthetic images with controlled properties (i.e., featuring a specific numerosity); on the other hand, they allowed studying the internal structure of the Transformer’s latent space, in order to investigate whether it could embed the semantics of cardinal numbers.

As an initial assessment, the Transformer was evaluated in a straightforward *conditional generation* task: given the ground-truth (x,n) tuple, the goal is to approximate x through the modeled $q(x|n,\theta^{⋆})$, incrementally building the image $\tilde{x}$ according to:

(12) $${\tilde{x}}_{i}=\arg\max_{x}q\left(x|x_{1},\ldots ,x_{i-1},n,\theta^{⋆}\right),\;\forall i\in \left[1,r\right].$$

In other words, each pixel is determined by those preceding it in the fixed scan order, and the current ground-truth pixel values are provided as input at each time step. This task was only used to monitor learning progress, since it is well-known that one-step-ahead prediction is much easier than autoregressive self-generation [38].

In the more challenging *spontaneous generation* tasks, the Transformer was required to build an entire novel image $\tilde{x}$ from scratch according to:

(13) $${\tilde{x}}_{i}∼q\left(x|{\tilde{x}}_{1},\ldots ,{\tilde{x}}_{i-1},n,\theta^{⋆}\right),\;\forall i\in \left[1,r\right].$$

Unlike (12), each pixel intensity is now conditioned on the previously sampled ones; Equation (13), therefore, requires *r* forward passes for each image. It should be noted that, during the first generative step, the encoder input sequence contains only the SoS. Carrying on, the sequence gradually incorporates the new intensity embeddings: $s^{T}$, ${\left[s,x_{1}^{T}\right]}^{T}$, ..., ${\left[s,x_{r-1}^{T}\right]}^{T}$, where the rows of $x_{i}$ correspond to the first *i* sampled gray levels. For each SoS considered, a fixed number of 64 images was generated, in order to collect statistics about the samples produced. Spontaneous generation was tested under four different conditions:

- *Spontaneous generation over trained numerosities.* In this case, the sampling process was initially conditioned on the |N| numerosity representations learned during the Transformer training (i.e., the rows of $W_{s}$). That is, the learned SoSs were provided as initial seed.
- *Spontaneous generation over interpolated numerosities.* In this case, we tested whether the generative process could be biased toward specific numerosities that were never encountered during training (but nevertheless fell in the training interval) by injecting a novel SoS as initial seed. Defining $w_{i}^{T}$ as the row of $W_{s}$ corresponding to the training numerosity *i*, the desired conditioning *n* is injected by simply setting $s=(w_{n-1}+w_{n+1})/2$. In other words, the new representation of *n* is linearly interpolated from the two closest SoSs.
- *Spontaneous generation over extrapolated numerosities.* The generative capability was further pushed by exploring whether the Transformer could be biased to produce numerosities falling outside the training range. The proposed extrapolation mechanism relies on the *attribute vector* technique described in [39], where the vector representing the direction of change is computed as $a=w_{\left|N\right|}-w_{|N|-1}$; it represents the direction along which the largest numerosities grow. We conjecture that the representation of a numerosity immediately larger than those included in the training range $\left[1,|N|\right]$ can be approximated by $s=w_{\left|N\right|}+\alpha a$, for a suitable α>0.
- *Spontaneous generation with reduced components.* Although the embedding size is constrained by the encoder architecture, numerosity information might in fact be mapped into a lower-dimensional space, akin to an ordered “number line” [40]. To explore the possibility that the learned SoSs could be arranged along a one- or two-dimensional subspace, we performed a principal component analysis (PCA) on the rows of $W_{s}$ and used either the first or the first and second principal components to reconstruct the SoSs used to start the generation process and thus establish whether the sampling quality is affected by such dimensionality reduction.

After all image pixels are generated, the number of dots produced needs to be estimated using a suitable heuristic. For the purpose, two dataset-specific heuristics were introduced. The first counter is a simple area-based heuristic designed to work with uniform dots-like samples. The generated numerosity, indeed, can be computed by simply dividing the area covered by the rendered dots in a frame by the average dot area; such mean value is trivially estimated from the validation split of the dots dataset. Since this heuristic does not work in the case of dots with different size, to estimate the number of dots produced by the Transformer trained on the *Non-Uniform Dots* dataset, a ResNet18 classifier [41] was employed. The ResNet18 was trained on a subset (22,000 samples) characterized by N={0,…,10}. Both counters achieved 100% accuracy on the respective dataset testing splits: the perfect accuracy achieved by the ResNet18 classifier on the test set suggests that numerosity estimation up to 10 items can be a trivial task for supervised deep learning models, at least with respect to the stimulus space considered in our simulations.

## 3. Results

After each generation task, the SoS-specific histograms of the generated numerosities were computed. We provide two different histogram visualizations: one depicts the relative frequency of each generated numerosity [34,42], while a 2D histogram is used to reproduce the visualization often used in human behavioral studies [43].

**Figure 3:**
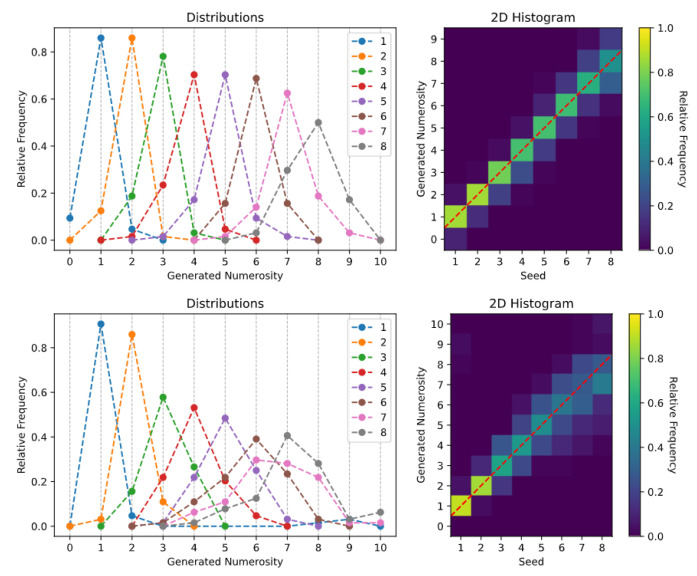
Spontaneous generation over trained numerosities: (**Top**) *Uniform Dots* dataset; and (**Bottom**) *Non-Uniform Dots* dataset.

**Figure A1:**
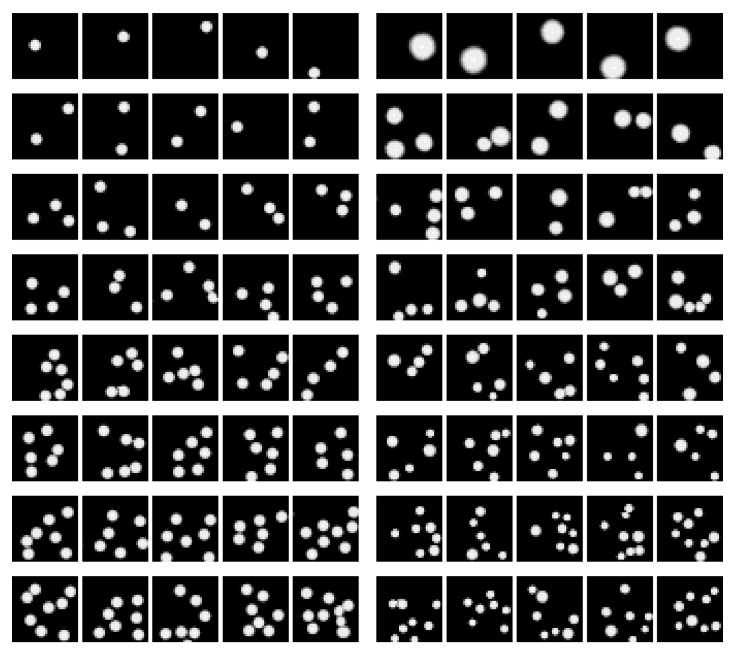
Sample images produced in the *spontaneous generation over trained numerosities* task: (**Left**) *Uniform Dots*; and (**Right**) *Non-Uniform Dots*. Images produced with increasing generation seeds (i.e., N={1,…,8}) are progressively shown from top to bottom. Sample images with a number of dots that does not match the seed are purposely included for illustration.

**Figure A2:**
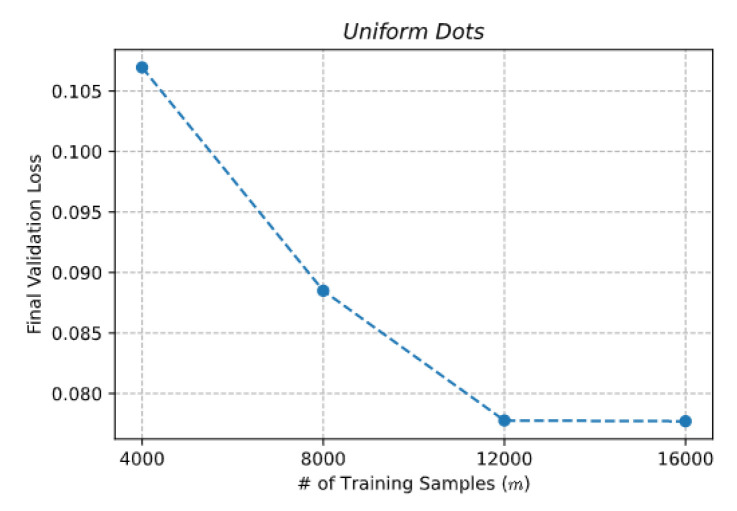
Converging validation loss obtained by training the Transformer (size “M”) on instances of the *Uniform Dots* dataset characterized by an increasing number of training patterns.

The generation histograms related to the *spontaneous generation over trained numerosities* task are shown in Figure 3. Especially for the *Uniform Dots* dataset (top panels), it is evident that the Transformer is able to create synthetic images with a specified numerosity, although the number of generated items is not always accurate. The generation is almost perfect for very small numbers (i.e., 1 and 2), while the model often generates one extra or one fewer item when asked to produce images with larger numerosities (see also the sample images reported in Figure A1). A similar pattern of errors is observed when the Transformer is trained using the *Non-Uniform Dots* dataset (bottom panels), although in this case the sampling uncertainty associated with larger numerosities increases, and the model sometimes generates images with a mismatch of up to three items. Overall, these results are well-aligned with the existing empirical literature on human behavior, which suggests that numerosity estimates are distributed around the target mean and variability tends to increase with numerosity [42,44], and that numerosity estimation can be altered by confounding non-numerical magnitudes [21,25].

**Figure A3:**
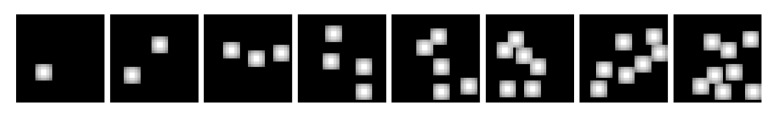
Sample images from the *Smoothed Squares* dataset. Stimuli with increasing numerosity (i.e., N={1,…,8}) are progressively shown from left to right.

**Figure A4:**
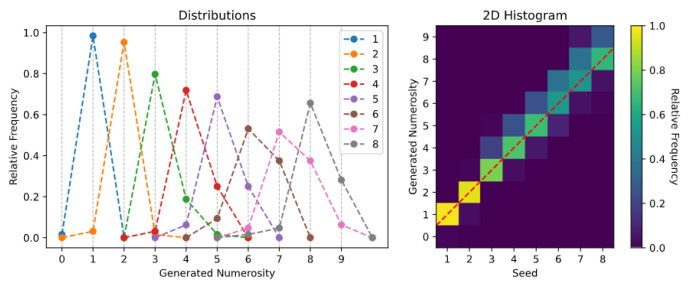
Spontaneous generation over trained numerosities on the *Smoothed Squares* dataset.

**Figure A5:**
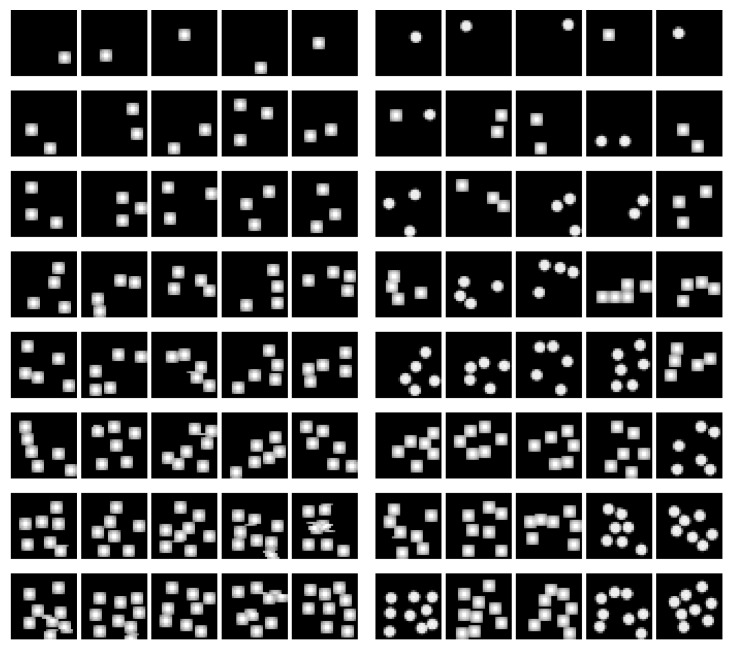
Sample images produced in the *spontaneous generation over trained numerosities* task by the model trained on *Smoothed Squares* (left) or a mix of *Smoothed Squares* and *Uniform dots* (right). Images produced with increasing generation seeds (i.e., N={1,…,8}) are progressively shown from top to bottom.

Notably, the synthetic images produced by the Transformer are much more precise compared to samples produced by other deep generative models, such as VAEs or GANs [26,34]. Moreover, differently from previous approaches, here we demonstrate that the generation process can be biased toward a specific numerosity, suggesting that attention mechanisms play a key role in allowing a more precise processing of numerosity information. As a control simulation, we also tested the generative capability of a model trained on images containing objects of a different shape. To this aim, we created the *Smoothed Squares* dataset containing images produced by inscribing squares into the circles of the *Uniform Dots* dataset, applying an average filtering (3×3) and a gamma correction ($x_{out}=x_{in}^{0.25}$). Sample images from this dataset are shown in Figure A3. Histograms related to the spontaneous generation task are shown in Figure A4, while samples of generated images are reported in the left panel of Figure A5. Interestingly, the Transformer generates well-formed images even when trained on a dataset containing a mix of images from *Smoothed Squares* and *Uniform Dots* (see the right panel of Figure A5), suggesting that it can properly factorize also shape information.

**Figure 4:**
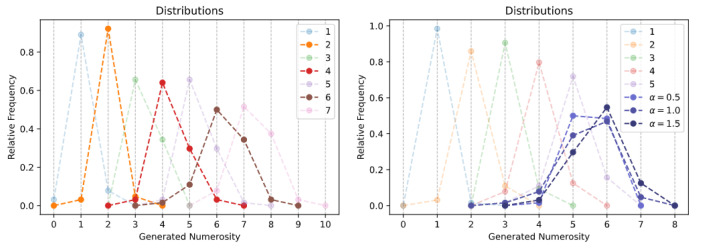
Spontaneous generation over interpolated (**left**) and extrapolated (**right**) numerosities. Trained numerosities are represented by semi-transparent curves, while solid curves represent unseen numerosities.

**Figure A6:**
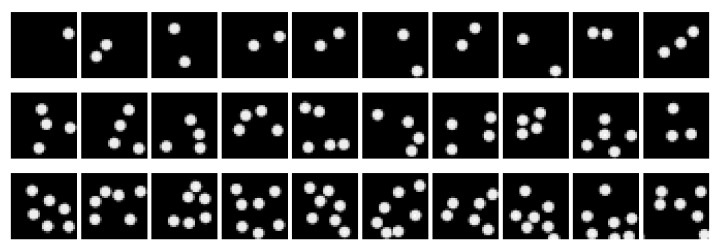
Sample images conditioned on the interpolated SoSs (i.e., *spontaneous generation over interpolated numerosities* task). From top to bottom, the displayed rows correspond to the unseen numerosities 2, 4 and 6, respectively.

The generation histograms related to the *spontaneous generation over interpolated numerosities* task are shown in the left panel of Figure 4. Quite impressively, the Transformer is able to produce images with a specific number of objects even for numerosities that were never encountered during training. For example, by averaging the embeddings corresponding to n=1 and n=3, the model always generates images with exactly two dots (orange line in the left panel). An analogous finding holds when interpolating the numerosities n=4 and n=6, although in those cases the number of items is not always perfectly matched (sample images are reported in Figure A6). These remarkable findings suggest that the emergent representational space approximately encodes the semantics of cardinal numbers, at least within the lower and upper training bounds.

**Figure A7:**
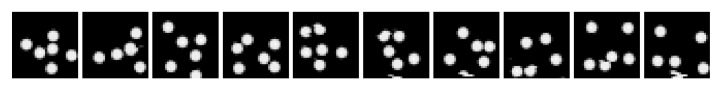
Sample images conditioned on the extrapolated SoS (i.e., *spontaneous generation over extrapolated numerosities* task). The results reported refer to α=1, which should produce images containing six objects. Note how the rendering of dots is qualitatively less precise than the ones shown in Figure A1 and Figure A6.

As shown in the right panel of Figure 4, the results related to the *spontaneous generation over extrapolated numerosities* task further corroborate this hypothesis. Indeed, the attribute vector computed as the difference between the embeddings of the two largest numerosities in the training set (in this case, n=4 and n=5) seems to represent the direction of increase of the numerosity feature: by summing a fraction of such vector to the embedding of n=5, the Transformer can reliably generate images containing six items, although sometimes the additional item appears squeezed or slightly distorted (sample images are reported in Figure A7). Interestingly, when the attribute vector is scaled by a factor alpha=0.5, the Transformer equally generates images with either five or six items. However, setting alpha≥2 did not allow to reliably generate images with seven items, suggesting that the learned embeddings approximately capture a sort of “successor function” only over the local neighborhood of a specific numerosity.

**Figure 5:**
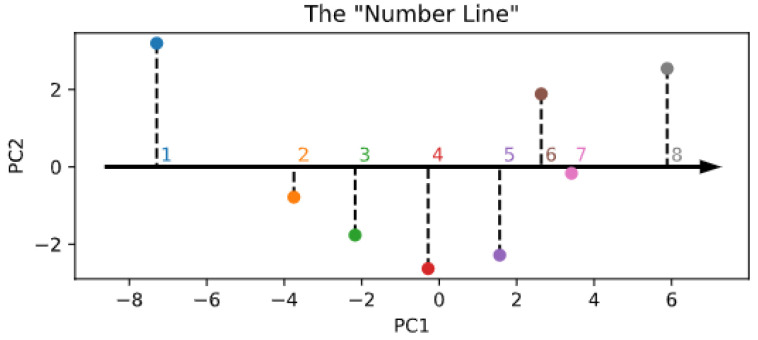
Visualization of the lower-dimensional embedding space resulting from PCA.

**Figure A8:**
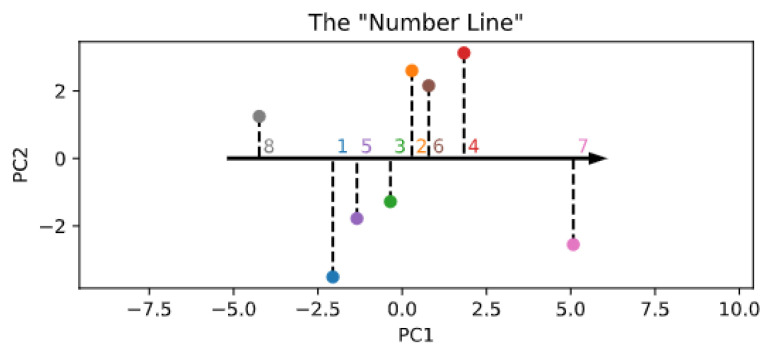
Visualization of the lower-dimensional embedding space right after random initialization.

The lower-dimensional manifold structure of the encoder space is shown in Figure 5. Interestingly, and in partial alignment with other recent computational work [35], it seems that the topology of the numerosity embeddings preserves the strict ordering of cardinal numbers, even though the Transformer did not explicitly receive such information during training. This is evident even by just looking at the first principal component (x-axis in the figure), which suggests that numerosity information could be internally organized as a one-dimensional “number line” [20,45]. However, differently from Kondapaneni and Perona [35], we found that the second principal component does not monotonically encode cardinal information, but suggests a periodic pattern. As a control analysis, Figure A8 also shows the PCA projection resulting right after the random initialization of the embeddings, which indeed does not reflect any ordering structure.

**Figure 6:**
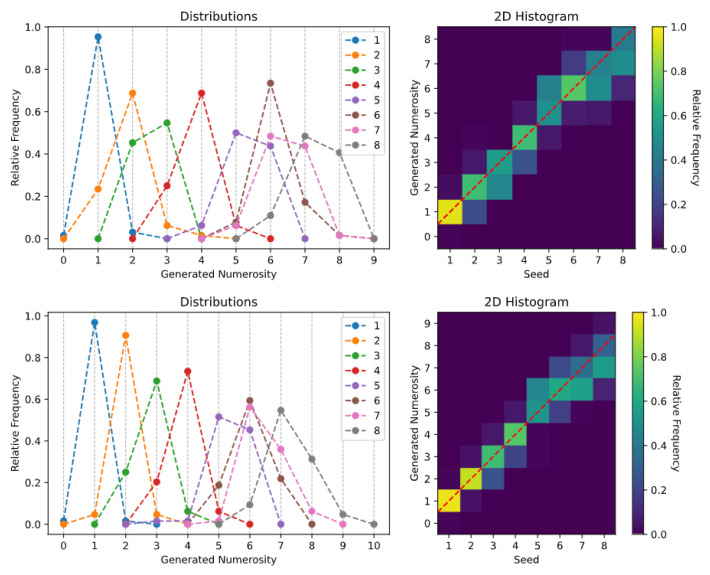
Spontaneous generation with reduced components, considering: one principal component (**top**); and two principal components (**bottom**).

The results related to the *spontaneous generation with reduced components* task suggest that, when the embeddings are projected into such lower-dimensional manifold, the generative abilities of the model are preserved: as shown in the top panels of Figure 6, the Transformer can generate samples with remarkable accuracy even when only the first principal component is retained. Adding the second principal component (bottom panels of Figure 6) allows further improving the generation precision, although numerosities mapped to nearby points in the lower dimensional space (i.e., n=6 and n=7) are frequently confounded.

## 4. Conclusions

In this study, we investigated whether state-of-the-art deep learning architectures based on attention mechanisms could learn disentangled representations of numerosity from a set of images containing a variable number of items. Our simulations not only show that Transformers can successfully learn to generate synthetic images featuring a target numerosity, but also that they can interpolate and extrapolate the generation process to previously unseen numerosities. These remarkable findings suggest that Transformers can indeed disentangle numerosity from other non-numerical visual features. However, it should be stressed that the generation process is error-prone and thus reflects an *approximate* representation of numerical information. Moreover, although we are impressed by the Transformer’s generative capabilities, in real world scenarios, the number of training patterns can be exponentially smaller than the support of the probability mass function to be estimated, which makes generalization to out-of-distribution samples particularly challenging [34]. A key open issue is thus to establish whether domain-general deep learning architectures could extrapolate numerical knowledge well beyond the limit of their training distribution, which would require learning more abstract conceptual structures, such as the successor function [46], which form the foundation of our understanding of natural numbers [47].

Another limitation of Transformer architectures is related to their computational complexity: naive implementations have a quadratic cost in the number of pixels in terms of both memory and computation, preventing their scaling to high resolutions. Recent studies have attempted to mitigate this issue by approximating global attention in different ways, for example by restricting self-attention receptive fields to local neighborhoods [31], reducing image resolution [32] or focusing on image patches [33]. In the present work, the image size allowed efficiently training and testing the Transformer architecture; however, future work should better clarify whether more effective attention mechanisms could be employed to scale-up the model to realistic image sizes.

## Data Availability

The source code for the simulations is available for download at https://github.com/BoCtrl-C?tab=repositories (accessed on 30 June 2021).

## Appendix A. Model Hyperparameters and Supplementary Figures

The Transformer described in the present work is available in three different sizes: “S” (∼37,000 parameters), “M” (∼136,000 parameters) and “L” (∼568,000 parameters); Table A1 reports the corresponding hyperparameters. For publication purposes, all presented results refer to the size “M” model. However, we also investigated the performance of the small and large variants on a subset of the introduced tasks, without noticing major differences. All models were trained using Adam optimizer [48] ($\beta_{1}=0.9$, $\beta_{2}=0.999$) with a batch size of 16. The training always stops when the validation loss does not improve, with respect to the best loss obtained, for five epochs in a row. After a search in the set {0.03,0.01,0.003,0.001}, the initial learning rate was set to 0.003. Furthermore, the learning rate decays by 0.1 every 25 training epochs. Each training session took, on average, 1 h 45 min on a workstation equipped with an NVidia GTX 1080 graphic card. To reduce the Transformer computational demand, the dataset entries were pre-processed by uniformly quantizing (16 levels) the input intensities (i.e., p=16).

**Table A1:** Hyperparameters characterizing the available model sizes. See the PyTorch Transformer documentation for more details about nhead and dim_feedforward (https://pytorch.org/docs/stable/generated/torch.nn.Transformer.html (accessed on 30 June 2021)).

Hyperparameter	Value		
	Size “S”	Size “M”	Size “L”
d_model (*d*)	16	32	64
nhead	2	2	4
num_encoder_layers (*L*)	6	8	10
dim_feedforward	64	128	256
